# *Candida auris* in Healthcare Facilities, New York, USA, 2013–2017

**DOI:** 10.3201/eid2410.180649

**Published:** 2018-10

**Authors:** Eleanor Adams, Monica Quinn, Sharon Tsay, Eugenie Poirot, Sudha Chaturvedi, Karen Southwick, Jane Greenko, Rafael Fernandez, Alex Kallen, Snigdha Vallabhaneni, Valerie Haley, Brad Hutton, Debra Blog, Emily Lutterloh, Howard Zucker, Candida auris Investigation Workgroup

**Affiliations:** New York State Department of Health, New Rochelle, New York, USA (E. Adams, K. Southwick);; New York State Department of Health, Albany, New York, USA (M. Quinn, S. Chaturvedi, V. Haley, B. Hutton, D. Blog, E. Lutterloh, H. Zucker);; Centers for Disease Control and Prevention, Atlanta, Georgia, USA (S. Tsay, E. Poirot, A. Kallen, S. Vallabhaneni);; New York City Department of Health and Mental Hygiene, New York, New York, USA (E. Poirot);; New York State Department of Health, Central Islip, New York, USA (J. Greenko);; New York State Department of Health, New York (R. Fernandez);; State University at Albany School of Public Health, Albany, New York, USA (V. Haley, D. Blog, E. Lutterloh)

**Keywords:** *Candida auris*, infection control, epidemiology, healthcare facilities, yeast, fungi, New York, United States

## Abstract

*Candida auris* is an emerging yeast that causes healthcare-associated infections. It can be misidentified by laboratories and often is resistant to antifungal medications. We describe an outbreak of *C. auris* infections in healthcare facilities in New York City, New York, USA. The investigation included laboratory surveillance, record reviews, site visits, contact tracing with cultures, and environmental sampling. We identified 51 clinical case-patients and 61 screening case-patients. Epidemiologic links indicated a large, interconnected web of affected healthcare facilities throughout New York City. Of the 51 clinical case-patients, 23 (45%) died within 90 days and isolates were resistant to fluconazole for 50 (98%). Of screening cultures performed for 572 persons (1,136 total cultures), results were *C. auris* positive for 61 (11%) persons. Environmental cultures were positive for samples from 15 of 20 facilities. Colonization was frequently identified during contact investigations; environmental contamination was also common.

*Candida auris* is an emerging yeast that has caused healthcare-associated infections on multiple continents (*1*–*13*). The organism was first described in 2009 by Satoh et al. for a patient in Japan (*14*). In November 2016, Vallabhaneni et al. (*11*) reported cases in the United States. Identification of *C. auris* requires specialized laboratory techniques (*15*–*17*). It is often resistant to antifungal medications (*18*), causes invasive infections (*1*,*4*,*5*) and outbreaks (*8*,*10*), and has become endemic to hospitals in some parts of the world (*2*,*5*,*6*). Therefore, its detection in New York, USA, healthcare facilities is concerning. We describe an ongoing outbreak of healthcare-associated *C. auris* cases involving multiple healthcare facilities in New York City (NYC), New York, USA, during 2013–2017.

## Methods

### Definitions and Data Analysis

We defined a case-patient as a person for whom a culture was positive for *C. auris*. Clinical cases are those for which the culture was obtained to diagnose or treat disease; screening cases are those for which the culture was obtained for surveillance purposes. We defined contacts as persons who had an epidemiologic link to a case-patient in place or time. We included clinical cases reported by April 30, 2017. Because surveillance cultures of contacts are performed after an associated clinical case is reported, we included surveillance cultures that were collected by June 26, 2017, and had final results available by July 19, 2017, which enabled us to better approximate the number of screening cases associated with clinical cases described herein. Data were analyzed by using SAS 9.4 (SAS Institute, Cary, NC, USA) and Excel 2016 (Microsoft, Redmond, WA, USA).

### Case Finding and Investigation

In June 2016, the Centers for Disease Control and Prevention (CDC) issued an alert about *C. auris* (*19*), after which the New York State Department of Health (NYSDOH) issued an advisory (*20*) to inform healthcare facilities about the emerging pathogen and request that they notify NYSDOH of potential cases and forward suspected isolates to the New York state public health laboratory (Mycology Laboratory at Wadsworth Center, Albany, NY, USA). In November 2016, NYSDOH issued a follow-up advisory (*21*) requesting that laboratories query their records for isolates of *C. auris* or species that could be confused with *C. auris*, such as *C. haemulonii*. Cases were also identified through active surveillance methods, including direct outreach to healthcare facilities and laboratories. Each clinical case was investigated through medical record reviews, contact tracing, and screening of contacts for colonization. 

### Contact Tracing

We obtained the names of persons who had resided in the same room as a case-patient in the 90 days before diagnosis for the case-patient. When close contacts could be located, we attempted to obtain samples for culture. 

### Surveillance and Infection Control Assessments

To emphasize the importance of detection, assist with infection control efforts, and conduct point prevalence surveys of facility contacts, we conducted site visits to facilities where transmission was suspected; our analysis included visits made through June 26, 2017. Initial point prevalence surveys included only a composite swab sample from the axilla and groin; subsequent surveys added a swab sample from the nares. For some persons, swab samples for surveillance cultures to identify persistent colonization were obtained and included samples from the axilla, groin, nares, rectum, wounds, and sites of noninvasive clinical infection. We also assessed key areas of healthcare infection control, including administrative support, hand hygiene, standard and transmission-based precautions, and environmental cleaning; we followed up with detailed assessments in specific areas as needed.

### Environmental Investigation

Whenever possible, we obtained samples from the environments of facilities where case-patients were admitted or resided. We concentrated on surfaces that were frequently touched and on objects in case-patients’ rooms.

### Laboratory Techniques

To isolate *C. auris* from patient screening swab and environmental specimens, we used the method described by Welsh et al., with slight modification (*15*). In brief, we used the ESwab Culture and Transport system (Becton Dickinson, Franklin Lakes, NJ, USA) and placed the samples in 1 mL liquid Amies transport medium. Samples were vortexed for 30 s, after which 50 μL was plated on nonselective Sabouraud dextrose agar containing antibacterials (SDA-A), 50 μL was plated on selective media including SDA-A enriched with 10% salt (SDA-AS), and 200 μL was transferred to 5 mL of Sabouraud dextrose broth containing antibacterials and 10% salt (SDB-AS). Later, for more selective recovery of *C. auris* from surveillance samples, we placed dulcitol, instead of dextrose, in the selective enrichment media.

We collected environmental samples by using sponge sticks (3M Health Care, St. Paul, MN, USA) and placed them in a zip-top bag containing 45 mL of phosphate-buffered saline (PBS) with 0.02% Tween 80. The bags were gently shaken for 1 min at 260 rpm in a Stomacher 400 Circulator (Laboratory Supply Network, Inc., Atkinson, NH, USA). The suspension without the sponge was poured in a 50-mL conical tube and centrifuged at 4,000 rpm for 5 min; supernatant was then decanted, leaving ≈3 mL of liquid in the bottom of the tube. We placed 50 μL of sponge suspension on different agar media and placed 1 mL of sponge suspension in 5 mL of SDB-AS broth, as we had done for patient swab samples.

Agar plates and broth tubes were incubated at 40°C for at least 2 weeks. To check for purity, we first streaked recovered yeast isolates on CHROMagar *Candida* medium (Difco; Becton Dickinson, Baltimore, MD, USA) and then subcultured them on SDA overnight and processed them for identification by matrix-assisted laser desorption/ionization time-of-flight (MALDI-TOF) mass spectrometry by using the standard ethanol–formic acid extraction procedure (*22*). Spectra were analyzed by using Flex Control 3.1 software (Bruker Daltonics, Inc., Billerica, MA, USA) and MALDI Biotyper OC version 3.1 (Bruker Daltonics, Bremen, Germany); per manufacturer’s instructions, a score of >2.0 was used to identify *Candida* to the species level. The in-house MALDI-TOF database was enriched by adding spectra from several *C. auris* isolates from the current outbreak and by adding reference isolates from the CDC AR bank (https://www.cdc.gov/drugresistance/resistance-bank/index.html); their identity was confirmed by DNA sequencing. To check for purity, we streaked the clinical isolates of yeasts received from healthcare facilities onto CHROMagar *Candida* medium and used MALDI-TOF mass spectrometry for identification as described.

The MICs of azoles and echinocandins were determined by using broth microdilution with custom TREK frozen broth microdilution panels (catalog no. CML2FCAN; Thermo Fisher Scientific, Marietta, OH, USA) (*23*). In brief, we prepared a suspension of *C. auris* at a concentration of 0.5 × 10^3^ to 2.5 × 10^3^ in RPMI–1640 medium (with glutamic acid and phenol red, and without bicarbonate; Sigma-Aldrich, St. Louis, MO, USA) and 0.2% glucose buffered to pH 7 with 0.165 mol/L 3-N morpholinepropanesulfonic acid (Sigma-Aldrich). We dispensed 100 μL *C. auris* inoculum into each well of the TREK plate. MICs of amphotericin B and 5-flucytosine were determined by Etest as recommended by the manufacturer (AB Biodisk; bioMérieux, Solna, Sweden) except that MICs were read at 24 h after incubation or until a confluent lawn of growth was seen. For Etests, the yeast inoculum was streaked on RPMI medium containing 2% glucose and 1.5% agar, and then E-test strips were applied. *C. krusei* (ATCC 6258) and *C. parapsilosis* (ATCC 22019) were used as quality control strains. The TREK broth and Etest plates were incubated at 35°C and read visually after 24 hours. Because there are no established *C. auris*–specific susceptibility breakpoints, we used tentative breakpoints published by CDC (*16*).

Environmental surveillance samples (sponges) were also processed by real-time PCR (*24*). In brief, 1 mL of sponge liquid was washed twice with PBS containing 0.1% bovine serum albumin; as an inhibition control, pellet was resuspended in 50 μL PBS with 0.1% bovine serum albumin containing bicoid plasmid. Each sample went through freezing, heating, bead-beating, and centrifugation, and 5 μL of extracted DNA was tested in duplicate by real-time PCR. According to receiver operator characteristic curve analysis, a cycle threshold value of <38 was reported as positive and >38 was reported as negative. If PCR inhibition was observed, specimens were reported as inconclusive. 

## Results

### Epidemiologic Investigation

**Figure 1:**
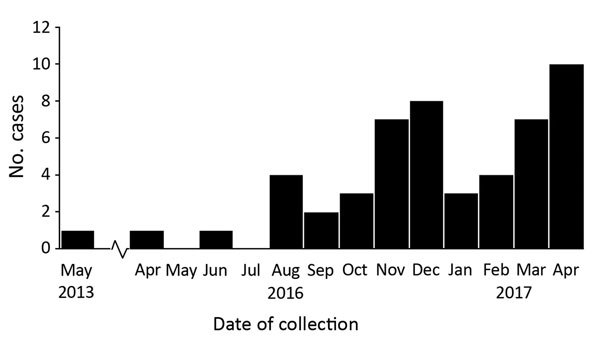
Number of confirmed clinical cases of *Candida auris* in New York, USA, May 2013–April 2017. Dates indicate the month that the first sample positive for *C. auris* was collected. The cases from May 2013, April 2016, and June 2016 were retrospectively identified after the June 2016 clinical alert from the Centers for Disease Control and Prevention was issued (*19*). The case from 2013, in a patient who had traveled to New York City from abroad for medical care, was probably a distinct importation with no further spread.

We detected 51 clinical and 61 screening cases (Figure 1). All but 1 of the clinical cases from New York were diagnosed in NYC: 21 from 7 hospitals in Brooklyn, 16 from 3 hospitals and 1 private medical office in Queens, 12 from 5 hospitals and 1 long-term acute care hospital in Manhattan, and 1 from a hospital in the Bronx. One clinical case was identified in a western New York hospital in a patient who had recently been admitted to an involved Brooklyn hospital. Of the 51 clinical case-patients, 31 (61%) had resided in long-term care facilities (LTCFs) immediately before being admitted to the hospital in which their infection was diagnosed, and 19 of these 31 resided in skilled nursing facilities with ventilator beds (VSNFs); 1 (2%) resided in a long-term acute care hospital; 5 (10%) had been transferred from another hospital; and 4 (8%) had traveled internationally within 5 years before diagnosis.

**Figure 2:**
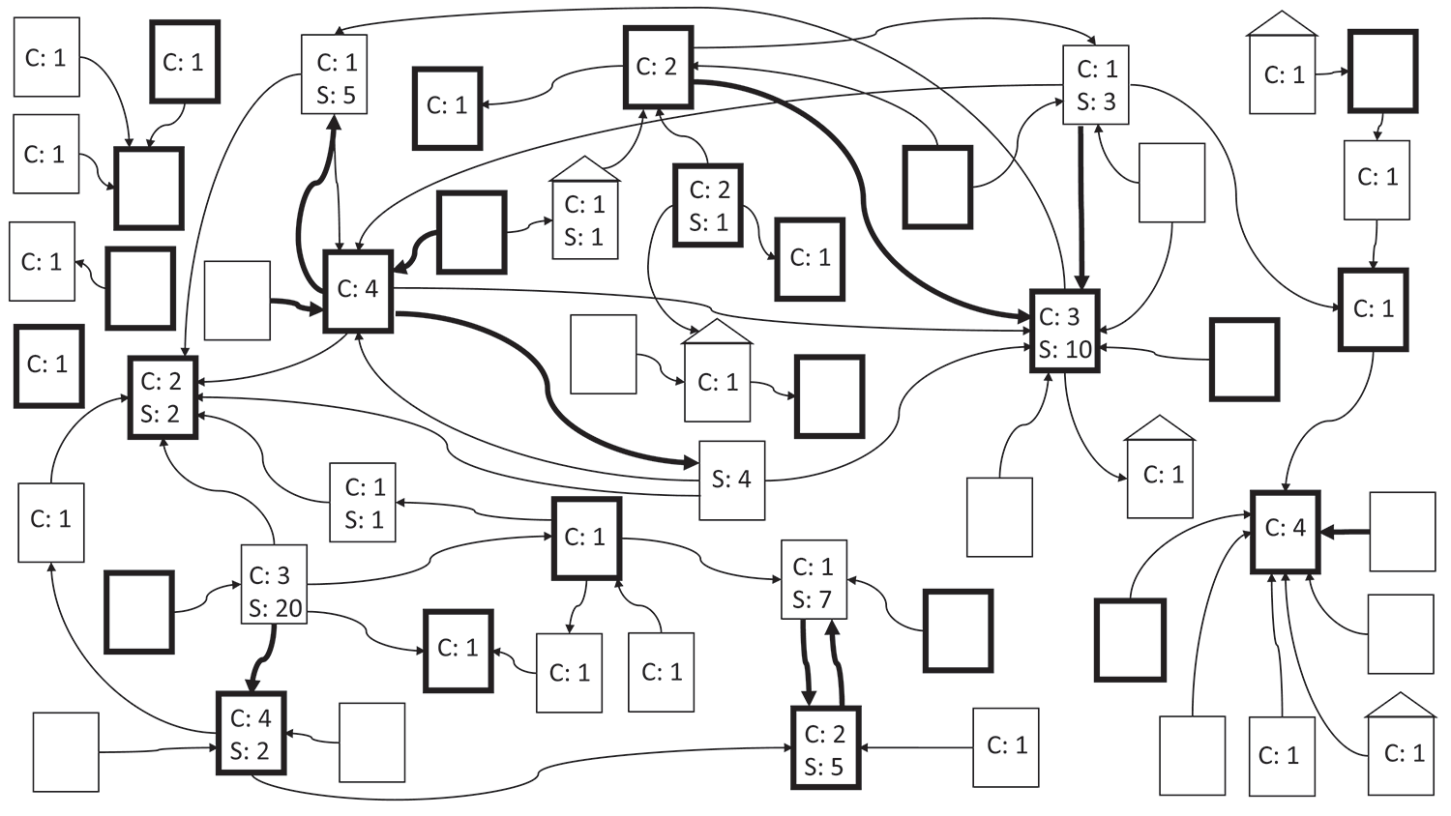
Epidemiologic links between healthcare facilities affected by *Candida auris*, New York, USA, 2013–2017. Arrows between facilities denote transfer of case-patients from one facility to the other within 90 days before collection date of first positive culture. Bold arrows indicate transfer of >1 case-patient. Bold boxes indicate hospitals; nonbold boxes indicate long-term care facilities; boxes with roofs indicate private residences. Numbers indicate numbers of clinical cases (C) and screening cases (S) at that facility. Screening cases are placed at the facility of diagnosis. Clinical cases are also shown at the facility of diagnosis unless the specimen was collected during the first week of admission at the diagnosing facility; in such situations, the cases are shown at the previous facility.

Exploration of epidemiologic links revealed a large, interconnected web of affected healthcare facilities throughout NYC (Figure 2). Determining the facility of acquisition of *C. auris* infection or colonization was difficult or impossible because of multiple healthcare exposures and because the incubation period is unknown.

### Clinical Characteristics

The median age of clinical case-patients was 72 years (range 21–96 years); 26 (51%) were male. All patients had serious concurrent medical conditions; a substantial proportion required mechanical ventilation or central venous catheters or gastrostomy tubes (Table 1). Initial positive cultures were from blood (31/51, 61%), bile (3/51, 6%), urine (4/51, 8%), respiratory specimens (4/51, 8%), wounds (3/51, 6%), catheter tips (2/51, 4%), and 1 each from bone, ear, jejunal biopsy sample, and skin. The 30-day mortality rate was 14/51 (27%), and the 90-day rate was 23/51 (45%). For those with initial isolates from blood, the 30-day mortality rate was 12/31 (39%) and the 90-day rate was 18/31 (58%). The number of deaths attributable to *C. auris* infection is unknown.

**Table 1:** Selected concurrent medical conditions and medical interventions for 51 persons with *Candida auris* infection, New York, USA, 2013–2017

Characteristic	No. (%) persons
Concurrent condition	
Respiratory insufficiency requiring support	33 (65)
Mechanical ventilation at time of diagnosis	17 (33)
Neurologic disease*	24 (47)
Diabetes	18 (35)
Malignancies	11 (22)
Colon cancer	5 (10)
End-stage renal disease	8 (16)
Hemodialysis	7 (14)
Kidney transplant	1 (2)
Decubitus ulcers	10 (20)
Otitis with complications	2 (4)
Medical interventions	
Hemodialysis	7 (14)
Central venous catheter within 7 d before first positive culture for *C. auris*	31 (61)
Gastrostomy tube at time of diagnosis	27 (53)
Receipt of systemic antifungal medication within 90 d before first culture positive for *C. auris*	25 (49)
Receipt of systemic antibiotics within 14 d before first culture positive for *C. auris*	42 (82)

*Includes seizure disorder (n = 8), cerebrovascular accident (n = 7), dementia (n = 4), anoxic brain injury (n = 3), spinal cord injury (n = 2), and 1 case each of Parkinson’s disease, multiple sclerosis, Huntington’s disease, Guillain-Barré syndrome, traumatic brain injury, pituitary tumor, and neuropathy.

### Surveillance Cultures

As part of point prevalence surveys and contact investigations, we performed 1,136 screening cultures for *C. auris* colonization for 572 persons not known to be infected and who resided in or were admitted to 19 facilities (9 hospitals; 10 LTCFs, of which 7 were VSNFs), 4 healthcare workers, and 4 family members of 1 clinical case-patient. At least 1 culture was positive for *C. auris* for 61 (11%) persons at 12 (60%) facilities (5 hospitals and 7 LTCFs [including 5 VSNFs]) and 1 family caregiver at a private residence. At the time of sample collection, 19 (31%) of these 61 persons were admitted to hospitals and 42 (67%) resided at LTCFs (40 [66%] at VSNFs). Culture results were positive for 13% of those who were tested while living at LTCFs and 8% of those in hospitals. 

**Figure 3:**
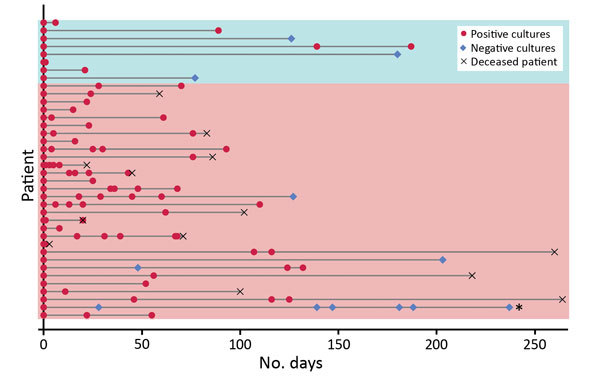
Long-term *Candida auris* colonization of clinical and screening case-patients, New York, USA, 2013–2017. Each patient for whom follow-up cultures were performed is represented by a horizontal line. The bottom 30 lines (pink shading) indicate clinical case-patients; the top 8 (blue shading) indicate screening case-patients. Follow-up cultures were collected from a variety of sites, typically axilla and groin and often nares, rectum, urine, and wounds. Persons were considered free of colonization with *C. auris* and eligible for removal of contact precautions when 2 sets of surveillance cultures at multiple sites, taken at least 1 week apart, were negative; only 1 person indicated on the figure (second from bottom) met this criterion.

For 38 persons (clinical and screening case-patients), follow-up cultures were performed, either for clinical reasons or to determine whether they remained colonized. Long-term colonization was common (Figure 3).

Of 346 persons for whom nares and composite axilla–groin samples for culture were collected on the same day, results were positive for at least 1 site for 36 (10%). Of these 36, results were positive for both sites for 14 (39%), at axilla–groin only for 13 (36%), and at nares only for 9 (25%).

### Environmental Cultures and PCR

Of 781 environmental samples from 20 facilities (12 hospitals and 8 LTCFs [5 VSNFs]), 62 (8%) from 15 facilities (9 hospitals and 6 LTCFs [4 VSNFs]) were positive for *C. auris* by culture. In addition, 19 samples from 4 facilities were positive by PCR; culture results for 3 of these 4 facilities were also positive. Contamination of surfaces and objects in case-patients’ rooms and mobile equipment outside the rooms was common (Table 2). High-yield items included bedrails, IV poles, beds, privacy and window curtains, windows, and floors.

**Table 2:** Environmental contamination with *Candida auris* in healthcare facilities, New York, USA, 2013–2017*

Category, object or surface	No. samples	Positive by culture, no. (%)	Positive by PCR and negative by culture, no. (%)	Negative by culture and PCR, no. (%)
Near-patient surfaces and objects in rooms				
Bedside/over bed table	44	2 (5)	2 (5)	40 (91)
Bed rail	49	7 (14)	5 (10)	37 (76)
TV remote/call button	36	2 (6)	2 (6)	32 (89)
IV poles	21	5 (24)	1 (5)	15 (71)
Bed	17	4 (24)	0	13 (77)
Privacy curtain	6	2 (33)	0	4 (67)
Miscellaneous other†	5	0	1 (20)	4 (80)
Total	178	22 (12)	11 (6)	145 (82)

Other surfaces and objects in rooms				
Door knob/handle	36	1 (3)	1 (3)	34 (94)
Sink	27	1 (4)	2 (7)	24 (89)
Window	22	3 (14)	1 (5)	18 (82)
Floor	17	4 (24)	0	13 (77)
Furniture	27	3 (11)	0	24 (89)
Window curtain	11	3 (27)	0	8 (73)
Light switch	9	0	0	9 (100)
Closet	6	0	0	6 (100)
Wall	4	1 (25)	0	3 (75)
Bathroom	4	1 (25)	0	3 (75)
Countertop	4	1 (25)	0	3 (75)
Toilet	4	0	0	4 (100)
Miscellaneous other‡	16	2 (13)	0	14 (88)
Total	187	20 (21)	4 (2)	163 (87)

Equipment in room				
Ventilator/respiratory equipment	12	1 (8)	0	11 (92)
Pump	4	0	0	4 (100)
Miscellaneous other§	19	4 (21)	0	15 (79)
Total	35	5 (14)	0	30 (86)

Equipment outside of room				
Clean supply cart	51	1 (2)	0	50 (98)
Ventilator/respiratory equipment	45	1 (2)	0	44 (98)
Vital sign machine	21	3 (14)	1 (5)	17 (81)
Normothermia system (e.g., Bair hugger)	20	1 (5)	0	19 (95)
Computer workstation	20	0	0	20 (100)
Thermometer	14	1 (7)	1 (7)	12 (86)
PPE/isolation cart/box	12	1 (8)	1 (8)	10 (83)
Lift/scale	11	2 (18)	0	9 (82)
Glucometer	11	0	0	11 (100)
Housekeeping cart	9	0	1 (11)	8 (89)
Dialysis equipment	7	1 (14)	0	6 (86)
Suction canister	6	1 (17)	0	5 (83)
Ultrasonography equipment	4	0	0	4 (100)
Miscellaneous other¶	29	1 (3)	0	28 (97)
Total	260	13 (5)	4 (2)	243 (94)

*A total of 660 samples were collected from surfaces, objects, and equipment in the rooms of *C. auris* case-patients and from mobile equipment outside the rooms on the affected nursing units. In addition, 62 samples from surfaces within the nursing units but outside the patient rooms and 23 samples from outside the affected nursing units were negative by culture and PCR. The location of 36 samples could not be ascertained; 2 were positive by culture. PPE, personal protective equipment; TV, television. †PCR positive from light cord. ‡Cultures positive from handrail and phone. §Cultures positive from glucometers (n = 2), vital signs machine, and stretcher. ¶Culture positive from bedpan flusher.

### Laboratory Identification of Clinical Isolates

From July 2016 through April 30, 2017, NYSDOH received 99 first isolates of a variety of yeasts from 99 persons, which clinical laboratories had sent for testing for *C. auris* (Table 3). Of those, 51 (52%) isolates were determined to be *C. auris* and represent the 51 clinical cases. Of the 99 isolates, 38 had been initially identified by the clinical laboratory as *C. haemulonii*, but NYSDOH determined 35 of those to be *C. auris*. Of 13 yeasts received with no identification, 11 were determined to be *C. auris*, and of 6 received with a preliminary identification of *C. auris, 5* were confirmed as such.

**Table 3:** Isolates received by the New York State public health laboratory, Wadsworth Center, Albany, NY, USA, from clinical laboratories for the purpose of identifying or excluding *Candida auris*, through April 30, 2017*

Organism, no. isolated	Clinical laboratories’ identification system				Wadsworth Center identification using MALDI-TOF MS, no. isolates‡
	API	VITEK 2	VITEK MS†	Other	
*Candida haemulonii*, 38		36		1§	*C. auris*, 35
				1¶	*C. haemulonii*, 1; *Candida duobushaemulonii*, 1; *Candida glabrata*,1
No identification, 13	2	2	9		*C. auris*, 11
					*C. glabrata*, 2
*C. auris*, 6				6§	*C. auris*, 5
					*C. duobushaemulonii*, 1
*Candida famata*, 5	1	3		1#	*C. guilliermondii*, 1; *C. lusitaniae*, 1; *Candida parapsilosis*, 2; *Candida fermentati*, 1
*Candida glabrata*, 1		1			*C. glabrata*, 1
*Candida guilliermondii*, 1		1			*C. guilliermondii*, 1
*Candida lusitaniae*, 1		1			*C. lusitaniae*, 1
*Candida sphaerica*, 1		1			*Saccharomyces cerevisiae*, 1
*Cryptococcus laurentii*, 1		1			*Cryptococcus neoformans*, 1
*C. neoformans*, 1		1			*S. cerevisiae*, 1
*C. famata/C. guilliermondii*, 1		1			*C. parapsilosis*, 1
*C. famata/C. parapsilosis*, 1		1			*C. parapsilosis*, 1
*C. famata/C. parapsilosis/* *Candida tropicalis*, 1		1			*C. parapsilosis*, 1
*Candida dubliniensis/C. haemulonii*, 1		1			*C. glabrata*, 1
*C. lusitaniae/Candida utiliz*, 1			1		*C. lusitaniae*, 1
*Candia sphaerica/Rhodotorula glutinis/ Rhodotorula mucilaginosa/C. laurentii*, 1		1			*C. parapsilosis*, 1
*Zygosaccharomyces bailii/C. sake/C. famata/Candida lipolytica*, 1		1			*C. glabrata*, 1
*S. cerevisiae*, 23	7	16			*S. cerevisiae*, 23
*Trichosporon mucoides*, 1		1			*T. mucoides*, 1

*API, bioMérieux, Inc., Durham, NC, USA; MALDI-TOF MS, Bruker Daltonics, Inc., Billerica, MA, USA; VITEK 2, bioMérieux, Durham, NC, USA; VITEK MS, bioMérieux, Solna, Sweden. MALDI-TOF, matrix-assisted laser desorption/ionization time-of-flight; MS, mass spectrometry.  †No *C. auris* entry. ‡Research use only library was expanded in-house by adding 10 *C. auris* isolates comprising clades I–IV (CDC-AR bank, https://www.cdc.gov/drugresistance/resistance-bank/index.html) and 8 *C. auris* isolates from New York in 2016 comprising clades I and II. §MALDI-TOF MS, research use only library with 3 *C. auris* entries. ¶BD Phoenix Automated Microbiology System (BD Diagnostics, Sparks, MD, USA). #RapID YEAST PLUS System (Thermo Fisher Scientific, Waltham, MA, USA).

### Susceptibility to Antifungal Medications

Of 51 initial *C. auris* isolates recovered from clinical case-patients, 50 (98%) were resistant to fluconazole (Table 4) and 13 (25%) were resistant to fluconazole and amphotericin B. No initial isolates were resistant to echinocandins, although subsequent isolates obtained from 3 persons who had received an echinocandin acquired resistance to it. According to whole-genome sequencing at CDC, 50 (98%) of 51 isolates belonged to a South Asia clade (*25*); the other less related isolate was the only isolate susceptible to fluconazole.

**Table 4:** Antifungal susceptibility data for first *Candida auris* isolates from 51 clinical cases, New York, USA, 2013–2017*

Antifungal	Tentative resistance breakpoint (*16*)	MIC_50_†	MIC range†	No. (%) resistant
Fluconazole	>32	>256	8.00 to >256	50 (98)
Itraconazole	NA	0.500	0.25–1.00	NA
Voriconazole	NA	2.000	0.50–4.00	NA
Posaconazole	NA	0.250	0.12–0.50	NA
Isavuconazole	NA	0.500	0.25–2.00	NA
Caspofungin	>2	0.060	0.03–0.25	0
Micafungin	>4	0.120	0.06–0.25	0
Anidulafungin	>4	0.250	0.12–0.50	0
Amphotericin B	>2	1.500	0.50–4.00	15 (29)
Flucytosine	NA	0.125	0.125–0.25	NA

*NA, not available. †MICs for azoles and echinocandins are defined as the lowest drug concentration that caused 50% growth inhibition compared with the drug-free controls; MICs for amphotericin B and flucytosine are defined as the lowest concentration at which there was 100% growth inhibition. MIC_50_ was defined as the MIC at which >50% of the isolates of *C. auris* tested were inhibited.

### Infection Control 

Infection control assessments were conducted at 14 LTCFs and 12 hospitals affected by *C. auris*. Adherence to recommended infection control practices, such as implementation of contact precautions, varied. Specific observations were made in the areas of hand hygiene, contact precautions, use of personal protective equipment (PPE), and environmental cleaning and disinfection.

Hand hygiene problems included frequent suboptimal availability of alcohol-based hand sanitizers. Sanitizers were completely absent in 1 LTCF.

A common problem with implementation of contact precautions was ineffective signage. One facility had no signs or other effective systems to identify persons around whom contact precautions should be taken. Compliance with signs that consisted only of instructions to see the nurse before entering was poor. In one instance, a physician entered a room with such a sign and provided care without donning PPE; when questioned, he stated, “I don’t see an isolation sign.”

Problems with PPE use included lack of knowledge about which PPE was indicated, improper donning and doffing (e.g., gowns not covering shoulders or not being tied), and lack of availability of appropriate PPE. In 1 LTCF, PPE was locked in a closet; in another, the PPE carts were empty and staff were unable to locate supplies to replenish them; in a third, aprons were used instead of gowns.

Environmental cleaning and disinfection observations included use of household cleaners instead of Environmental Protection Agency–registered hospital-grade disinfectants (at some LTCFs), use of disinfectants without appropriate label claims, inadequate disinfection of shared equipment, and lack of knowledge of contact times.

## Discussion

This large, citywide, multiple-institution outbreak of *C. auris* infections is ongoing. As of February 2018, most confirmed clinical cases in the United States had been identified in New York, and case numbers continue to grow. The reasons for the preponderance of cases in New York are unknown; possibilities include a true higher prevalence from multiple introductions into this international port of entry, more complete detection from aggressive case finding, presence of a large interconnected network of healthcare facilities in NYC, or a combination of all 3 factors.

Transmission is ongoing in healthcare facilities, primarily among patients with extensive healthcare exposures. *C. auris* has been cultured from rooms and equipment in multiple facilities, and close contacts of case-patients have become colonized. Infection control lapses have probably amplified this process.

Factors that contribute to transmission may include prolonged colonization of clinical and screening case-patients and environmental contamination. Persistent colonization of affected persons and the lack of an accepted decolonization regimen result in a large reservoir of persons carrying the organism. As Welsh et al. (*15*) demonstrated, *C. auris* can remain viable on inanimate surfaces for long periods, necessitating scrupulous environmental cleaning and disinfection. Affected patients frequently have extensive contact with multiple healthcare facilities, highlighting the value of careful and thorough communication at transfer.

The clinical cases in the New York outbreak are similar to clinical cases described elsewhere. Fungemia is a commonly reported clinical infection; 76% of infections reported in a series from Colombia (*9*) and 58% in a series from India (*4*) were bloodstream infections. These percentages are comparable to the findings from this New York series in which 61% of initial clinical isolates were from blood. Among medically fragile patients in NYC who had a history of extensive contact with healthcare facilities, clinicians should include *C. auris* in the differential diagnosis for patients with symptoms compatible with bloodstream infection.

Limitations of this investigation include the inability to determine where *C. auris* was acquired for most cases because of multiple healthcare exposures. Point prevalence surveys have not yet been conducted at all involved facilities. The best uses for and interpretation of PCR results are still being determined, especially when PCR is positive but culture result is negative. This investigation did not assess transmission in the community or outpatient settings; other investigators have described *C. auris* infections associated with an outpatient clinic (*12*).

Given the consequences of the development and spread of multidrug-resistant bacteria over the past several decades, the prospect of an endemic or epidemic multidrug-resistant yeast in US healthcare facilities is troubling. Data from other countries show that *C. auris* can become established within facilities. Chowdhary et al. (*2*) report that *C. auris* accounted for 5% of candidemia cases in a pediatric hospital and 30% in a tertiary general hospital in India. Chakrabarti et al. (*6*) report that *C. auris* was isolated from 19 of 27 intensive care units throughout India and accounted for 5.2% of *Candida* isolates from intensive care units. Okinda et al. (*26*) report that 38% of candidemia cases at a referral hospital in Africa were caused by *C. auris*, surpassing *C. albicans* cases (27%). Schelenz et al. (*10*) describe an outbreak in a London hospital that affected 50 patients.

Infection control lapses observed during site visits prompted NYSDOH leadership to conduct educational webinars for New York state clinicians and onsite infection control–focused reviews of all nonfederal hospitals and LTCFs in Brooklyn and Queens. NYSDOH also created a web page for healthcare personnel and the public (*27*). Intensive infection prevention and control efforts continue; the goals are delaying endemicity, preventing outbreaks within facilities, reducing transmission and geographic spread, and blunting the effect of *C. auris* in New York and the rest of the United States.
